# Ecological footprints, global sustainability, and the roles of natural resources, financial development, and economic growth

**DOI:** 10.1371/journal.pone.0317664

**Published:** 2025-03-13

**Authors:** Ali Hussein A. L. Marshadi, Muhammad Aslam, Azhar Ali Janjua

**Affiliations:** 1 Department of Statistics, Faculty of Science, King Abdulaziz University, Jeddah, Saudi Arabia; 2 Department for Higher Education, Deputy Director Colleges Hafizabad, Punjab, Pakistan; Zhejiang Sci-Tech University, CHINA

## Abstract

The prevailing ecological deficit is devastating the ecosystem which is leading toward the unsustainability by endangering the livings on earth. The important drivers of this environment degradation are natural resources depletion, financial development and the economic growth which are investigated to test their impact on ecological footprints. The EKC hypothesis is evaluated to test the growth led environment pattern. This study incorporated twenty years (2002–2021) data of 146 countries and the group of countries are investigated into various quantiles, geographical regions and income wise groups formed by the World Bank. Keeping in view the heterogeneous data established through the Shapiro-Francia W test and graphical analysis, the panel quantile regression is used which is insensitive to heterogeneous data. Firstly, the impact of dependent variables is estimated on environment degradation using the 10^th^, 20^th^, 30^th^,40^th^, 50^th^, 60^th^, 70^th^, 80^th^, 90^th^ and 99^th^ quantiles. The results suggested that the natural resources depletion and financial development are deteriorating the ecological footprints, which varies from smaller in initial quantiles to large in later quantiles. NR in North America and FD in the Middle East & North Africa are severely deteriorating the environment quality. The economic growth is improving the ecology in East Asia and the Pacific. The negative impact of natural resources on environment degradation is found in all income groups albeit with varying intensities. The financial development in upper middle income and lower middle income groups is deteriorating the environment quality. The EKC hypothesis remained undetermined for the estimated quantiles and geographical regions whereas it is established in high income group only. The policy intervention is recommended to restrict the natural resources depletion and binding the credit facilities to invest in ecosystem friendly projects by curtailing the process of ecological deficit for global sustainability which may be initiated from the most environment degraded quantiles, geographical region and income group.

## 1. Introduction

Environmental constraints and scarcity of natural resources stimulated the concerns about sustainability by accentuating the maintenance of natural resources and the environment [1–4]. Ecological evaluation is significantly important for the effectiveness of sustainability [5] as the growth pattern accomplished by many economies is moving toward unsustainability [6]. Owing to rising environmental degradation the policy makers and researchers’ attention has turned towards the identification of factors causing that pollution [7]. The deteriorating environmental quality is worrisome for both developed and developing countries [8] which is one of the main causes of the emergence of the burning concern of sustainability. The rapid industrialization and uninterrupted extraction for utilization of natural resources deteriorated the quality of the environment [9]. Environmental degradation (ED) is multidimensional from water and air pollution to the reduction of ecological resources and more importantly, it is threatening the sustainability of the global economy as it is closely linked with numerous macroeconomic indicators [10]. Environmental degradation is linked with climate change which hampers the agricultural output and food supplies, it exerts adverse conditions for human health, economic activities, energy utilization, physical capital, productivity, and access to water [11]. Subsequently, significant environmental-led socio-economic concerns established the global movement to actively restrain the undesirable consequences.

In the year 2015, the member states of the United Nations (UN) proposed 17 Sustainable Development Goals (SDGs), targeted by 2030, to ensure social, economic and environmental sustainability for the peace and prosperity of the earth and its inhabitants. The failure to attain SDG 13, (i.e., climate actions) with SDG 7, (green and inexpensive energy) affecting in achieving SDG 8 (i.e., decent work and economic growth). SDGs (SDG 6, SDG 13, SDG 14 and SDG 15) necessitate a comprehensively integrated approach to address environmental contamination. UN vision-2030 states that ecological apprehensions and the SDGs are unachievable without improving the environmental concerns [5,9,12]. The global environmental degradation is not reverting to its mean behavior and the shocks have permanent effect thus the reduction in environmental degradation is possible if the policies are established in international agreement [13]. Almost 200 global countries endorsed the Paris Agreement which required submission of National Determined Contribution (NDCs) to the United Nations Framework Convention on Climate Change (UNFCCC) to achieve the mitigation targets of emissions of Greenhouse Gases (GHGs) to resist climate change concerns [14] The countries are submitting more focused pledges to reduce the GHG emissions to net zero by 2050 [15]. In recent years, environmental degradation has been up-surged from a low urgency to the utmost global issue. SDG Progress Report 2023 states that *“The world in on the brink of a climate catastrophe and current actions and plans to address the crisis are insufficient”*[16].

The need of cleaner and safer environment for the livings on earth remains an important issue and most of the ecological related investigations addressed the damage to environmental quality and the capability of nature to redevelop natural resources. Natural resources are becoming scarce with rapid economic growth and the extraction of natural resources may damage the environment by polluting water and land [17]. The focus remained on determining the root causes of pollution and the procedures for reducing it. Two proxies are used to enumerate environmental degradation, ecological footprint (ECF) and carbon dioxide (CO2) emission. CO2 is mostly used proxy for environmental degradation which envisages various socio-economic activities; however, it is recommended a weak indicator [18] inadequate to capture the influence of human activities on the ecosystem [19], whereas, it is a part of environmental degradation which is mainly the result of energy consumption [20,21]. ECF signifies the pressure on natural resources mainly due to population [22]. It is the burden of anthropogenic activities imposed on natural environments [21]. The comprehensive nature of ECF in capturing the impact of production and consumption urges us to use it as a proxy for environmental degradation [11,23]. Many research studies evaluated the impact in multiple dimensions which are affecting ECF, for example, investment and remittances [24,25], financial development [26,27], tourism [28], human capital [29], social welfare [30], energy consumption [27] and human health [31] which evident the interrelationship of nature and society.

**Fig 1:**
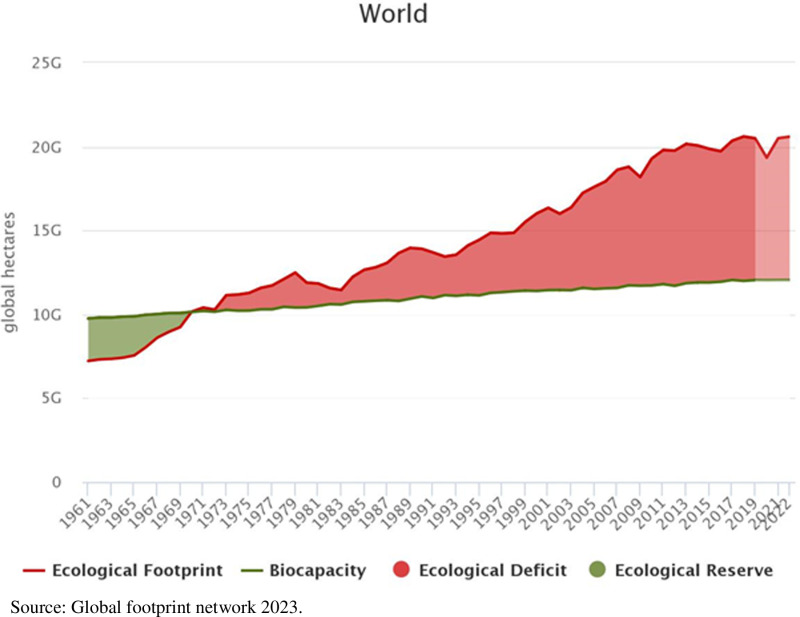
Global ecological footprints.

Fig. 1 depicts the global ecological footprints which shows the rising pattern of deficit from 1973. Environmental degradation emerged with the enervation of natural resources [32] as the utilization surpasses the productive capability of natural resources. The debate of green development has been emerged [33] which necessitates the investigation on consumption of natural resources. The environmental imbalance is damaging the bio-capacity of the earth and playing a critical role in the depletion of natural resources and the accumulation of GHGs while posing threats to all the living on the globe.

The rapid growth of economic activities is an important aspect of environmental degradation. This relationship can be well explained with the help of Environmental Kuznet Curve (EKC). The nexus between ED and economic growth was tested in the 1990s by incorporating the Environmental Kuznet Curve (EKC) theory [9]. EKC states an inverted U-shaped relationship between ED and economic growth. According to it, in the long run economic growth will not be a threat to the environment. To capture this impact GDP and square of GDP are taken into account [34]. Rafindadi [35] investigation Japanese dataset (1961–2012) which supported the existence of the EKC.

Economic growth, extraction of natural resources and technological innovations bears significant impact on ecological footprint. Machinery prices and investment for technological innovations may be used to minimize harm to the environment. Financial development (FD) is considered an imperative factor to purchase these advanced technologies so this study is incorporating FD as an important variable. A successful financial system works as an engine of growth by reducing the cost of doing business thus the FD aids in efficient utilization of resource with less depletion and pollution. Which results in less environmental degradation [7]. Opuala et al. [27] estimated that financial development and natural resource rent did not contribute for environmental quality in West Africa. Ding [36] explained that the development of financial system is necessary for the capital accumulation, economic growth and prosperity of any nation, however many resource abundant countries have lower level of FD and it may be a curse or blessings depends on its contribution to economic development, i.e., resource rents are positively associated with financial development [36]. Godil et al. [37] endorsed that the FD leads to improve efficiency by adopting the opportunity to use advanced technologies supportive to environmental concerns. Contrary to it, some studies [38,39] support FD as one of the reasons to enhance the ecological footprint. FD is used as a growth strategy by the policy makers to stimulate economic activities. FD is used for the expansion of markets, instruments, and financial institutes [40]. Every expenditure for construction, machinery or an automobile has an impact on the environment as this production entails energy consumption and emit pollutant which adversely impact the environment.

In recent years, environmental degradation has up-surged from a low urgency to the utmost global issue. In this regard, the vision-2030 of the United Nations (UN) states those ecological apprehensions and some SDGs have been well-known for the world. The SDGs are unachievable without improving the environmental concerns. Godil et al. [41] the sources of pollution differ among countries those depend on various driving forces. This study identifies the important drivers behind the emergence of the issues of sustainability. In this regard, this study explores the impact of natural resources, financial development and economic growth on environmental degradation by incorporating relative new variable, i.e., ecological footprint. Erdogan et al. [13] necessitated the investigation of environmental concerns in global perspective and this study contributes in literature by incorporating a unique comprehensive twenty years’ dataset of 146 countries across the globe. The dataset is investigated in multiple dimensions to extract the valuable information by bifurcating it, into income groups and geographical regional groups, using the criterions of World Bank. Then, the environmental Kuznets curve hypothesis is examined with respect to income groups and geographical region wise groups of countries. Keeping in view the heterogeneous nature of the dataset the quantile regression approach is applied to understand the behavior of the factors to enumerate the quantile wise influence on the environment degradation. The study investigated the behavior of the variables; region wise, income groups of the countries and the global perspective is presented. Therefore, the inspiring nature of this research is to contribute the thought provoking realistic approach to unhindered the relationship between the valued variables to formulate the helpful policies globally and for the countries regarding their income groups and geographical regions for effective decision making.

This study is organized as the section 1 presents the introduction, section 2 explains the relevant literature review, section 3 describes the methodology and data description, next the section 4 presents the results and discussion of these outcomes, section 5 is the conclusion including the policy implication and limitations, In the last references and annexures are presented respectively.

## 2. Literature review

Economics has been concerned with efficient utilization of natural resources. Adam Smith examined the land, mines; Ricardo explored the quality of land for determining the rent; Malthus pointed out the population, poverty and limits of agricultural resources. All these economists treated the natural resources as a factor of production gifted by nature. Invisible hands work for efficient distribution of resources for maximization of social welfare. In the last thirty years the emergence of environmental issues clarified that the extraction of natural resources is not utilized for maximization of social welfare and the market failure in calculating the cost of using natural resources has been emerged. Lee et al. [2] examined the natural resource and sustainability for the seven developed (G7) economies that found that the natural resources significantly reduce the ecological footprints. Erdogan et al. [33] explored the effect of fossil fuels consumption on environmental degradation in G7 countries using robust panel estimation and found that it dampens the environmental quality. Ding [36] examined the impact of natural resources on financial development in group of seven (G7) countries incorporating quantile regression on dataset (1990–2020) and found that the natural resources adversely affect the financial development while the bidirectional relationship also exist among these variables. Authoritative intervention is recommended for sustainable extraction of natural resources and to regularize the utilization of financial development [36]. Yang el. [42] pointed out the excessive mining, depletion of forests, required agricultural production and dependence of black economy for generation of income from natural resources endangered sustainability. Jie et al. [43] investigated the impact of natural resources, industrial development and population growth on energy sustainability and socio-economic development in China. The results explained a positive association between natural resources, economic growth and ecological footprint while long term socio-economic development leads to environmental sustainability. Xiong et al. [17] studied Chinese dataset from 1971 to 2018 and found that the environmental degradation and extraction of natural resources are interconnected and have significant impact on the livings on earth through soil erosion, deforestation and pollution. Governments must ensure to implement regulations to protect the environment and use the resources sustainably [17]. Rafindadi [44] investigated the South Africa’s environment degradation using time series dataset from 1971 to 2014 and found an upward dynamics linking the excessive usage of fossil fuel. Opuala et al. [27] inspected the ecological footprints in West Africa by taking the panel dataset 1980–2017 and incorporated the modified STIRPAT model and found the positive causality between natural resource rent to the ecological footprint however the financial development did not contribute to the environment quality. Kumar et al. [1] pointed out the growing exploitation of natural resources which is leading toward the depletion of nonrenewable natural resources in the near future. The rate of consumption is far more rapid than the regeneration process. Climate change and the growing population are threatening natural resources. The literature explained the excessive extraction of natural resources are stimulating the concerns and it may trigger further by deteriorating the environmental quality. The ready available studies are country specific or addressing the group of countries thus it is necessary to investigate the matter in a broader perspective.

Financial development (FD) is considered an imperative factor to purchase the advanced technologies for production and accumulation of wealth thus this study is incorporating FD as an important variable. Godil et al. [37] endorsed that the FD leads to improve efficiency by adopting the opportunity to use advanced technologies supportive to environmental concerns. Contrary to it, some studies [38,39] described FD as one of the reasons to enhance the ecological footprint as it is used as a growth strategy by the policy makers to stimulate economic activities. FD is used for the expansion of markets, instruments, and financial institutes [40] which adversely impact the environment. Every expenditure for construction, machinery or an automobile has an impact on the environment as this production entails energy consumption and emit pollutant. Godil et al. [7] investigated the impact of financial development on CO2 emission in Pakistan during 1995 to 2018 and found that the financial development has a negative impact on environment degradation. Rafindadi [45] investigated the impact of financial development on environmental degradation in Nigeria using time series data (1970–2011) and the findings revealed that the financial development lowers the environmental degradation. Rafindadi et al. [46] examine the impact of financial development in Saudi Arabia during 1971–2015 and found that the financial development aggravates the energy consumption which leads to environment degradation thus necessitated to influence the policy intervention to move toward the sustainability. Ding [36] explained the importance for developing the financial system for economic growth and prosperity, while many resource abundant countries have lower level of financial development and the financial development may be a curse or blessings which depends on the its contribution to economic development, i.e., resource rents are positively associated with financial development. The resource rich countries should focus on increasing technological innovation through financial development to the improve environment and economic condition [47]. FD is used as a growth strategy to stimulate the national income however the utilization of financial resources may determine its impact on environment. Acar et al. [48] elucidated an inverted U-shaped EKC between economic growth and ecological footprint and suggested that the financial development squeezes ecological footprint.

The nexus between ED and economic growth was tested in the 1990s by incorporating the environmental Kuznet Curve (EKC) theory [9] which states that the long run economic growth will not be a threat to the environment. Lee et al. [2] examined panel quantile regression for OECD countries and found a nonlinear relationship between economic growth and ecological footprint and an inverted U-shaped association endorsed the theory of EKC. Chekouri et al. [49] suggested that GDP per capita upsurges ecological footprint. Li et al. [3] explained that economic growth stimulated with nonrenewable energy leading to environmental degradation. Ansari [50] investigated EKC for Association of South East Asian Nations (ASEAN) countries by incorporating ecological footprint and estimated that the inverted U-shaped theory exists. Fossil fuels rich countries are not imposing the limit to haunt the demand for fossil fuels and compromising the environmental degradation [51]. The resource rich countries are recommended to improve the technological innovation to improve the environmental and economic condition [47]. Jahanger et al. [10] tested EKC for the African and Latin American, and Caribbean nations and suggested the developing economies implement policies relevant to them for efficient utilization of natural resources by using advanced technologies. It recommended adopting sustainable environmental objectives in making and implementing policies. Sarwar et al. [34] validates the EKC hypothesis for BRICS countries. Pata et al. [52] studied the role of technologies for curbing the environmental degradation by using the data (1989–2020) of four technological advanced countries and tests the validity of EKC. The investigation highlighted that the countries should rely on use of technologies and the income growth as the effective policy to mitigate environmental degradation. Godil et al. [7] supported the EKC hypothesis for the case of Pakistan using data from 1995 to 2018. Erdogan et al. [13] examined the shocks to global environmental degradation over 2000 years and concluded that the emissions does not reverting to its mean behavior and the external shocks to emissions have permanent effects thus suggested to establish effective environment policies globally. Rafindadi [45] used the time series data from 1971 to 2011 and found that the economic growth stimulated the environment degradation in Nageria. Rafindadi [35] investigated the Japanese EKC using dataset (1961–2012) which supported the economic growth and environment degradation having the Inverted-U shape relationship however 1% increase in real GDP leads to increase emission by 5.76%. Rafindadi [44] estimated an upward relationship between economic growth and environment degradation in South Africa during 1971–2014. The literature evident of mixed pattern of growth led environment relationship as described through EKC hypothesis.

## 3. Methodology

The classical regression is preferably used for the data following normal distribution. It considers all the data and does not ignore the outliers. When the assumptions of classical regression are violated, It overestimates or underestimates the coefficients of heterogeneous distribution [53]. Quantile regression for panel data was proposed [54,55] to overcome the limitations of classical regression. It is flexible for heterogeneous distribution and provides robust outcomes in the presence of outliers [56]. Panel data and quantile regression models are commonly used in theoretical and applied research. Quantile regression models permit the researcher to account for unobserved heterogeneity and heterogeneous covariates effects. The median regression ignores the outliers and results the better fit however median is also the single location. The median regression is a special case of quantile regression as it model’s other quantiles thus it estimates the desired level of segments of the dataset [57]. In Bootstrap the replications are used to estimate the variance covariance matrix of the estimators. Quantile regression for panel data is estimated using Stata-15 as suggested [58] which addresses the problem posed by alternative fixed effect quantile estimators [59]. Adaptive Markov Chain Monte Carlo (MCMC) sampling using a continually adapted multivariate normal proposal distribution described [60] is estimated. The structure of the quantile regression is presented in equation (1)


$$E_{yi}\left(\tau \text{|}x_{i}\right)=X_{i\beta \tau }^{t}$$ (1)

Following [61], this study used quantile regression which describes the impact of independent factors in different quantiles and estimates the impact of natural resource depletion, domestic credit to the private sector and economic growth on ecological footprints.


$$E_{t}\left(ED_{it}\text{|}X_{it}\right)=\theta_{t}+\beta_{t}X_{it}+\theta_{t}\varepsilon_{it}$$ (2)

The quantile τ^th^ (0 < τ < 1) conditional distribution of the dependent variable as a function of independent variables *X*_*it*_. While the $\varepsilon_{it}$ estimate the unobserved effects.


$$min_{\left(\theta ,\beta \right)}\overset{K}{\underset{k=1}{\sum }}\overset{T}{\underset{t=1}{\sum }}\overset{N}{\underset{i=1}{\sum }}W_{k}P_{tk}\left(y_{it}-\theta_{i}-X_{it}^{T}\beta_{\tau k}\right)+\gamma \overset{N}{\underset{l}{\sum }}\left|\theta_{l}\right|$$ (3)

The residuals are minimized using the equation (3). Where *i* shows the number of countries (N), *t* represents the time period, *K* indicates the quantiles, *X*_*it*_ is the set of explanatory variables, *P*_*tk*_ is the quantile loss function, *W*_*k*_ represents the weight assigned to the quantile loss function. *γ* indicates the adjustment parameter to improve the estimation of *β* and decrease the individual effect to zero.


$$E_{yi}\left(\tau \text{|}\theta_{i},\varepsilon_{t},X_{it}\right)=\theta_{i}+\varepsilon_{t}+\beta_{1\tau }NR_{it}+\beta_{2\tau }FD_{it}+\beta_{3\tau }EG_{it}+\beta_{4\tau }GS_{it}+\Phi_{it}$$ (4)

For further understanding of the methodology the listed literature may be consulted [2,58,61]. Environment degradation (ED), Natural resource (NR), Financial development (FD), Economic growth (EG) and Economic growth square (GS) are the variables of the study. The literature endorsed that economic growth, extraction of natural resources and technological innovations theory was tested the impact on environmental degradation. Machinery prices and investment for technological innovations may be used to minimize harm to the environment. Financial development (FD) is considered an imperative factor to purchase these advanced technologies so this study is incorporating FD as an explanatory variable.

Equation (4) represents the Environment Kuznet Curve (EKC) hypothesis which states an inverted U-shaped relationship between ED and economic growth. According to it, in the long run economic growth will not be the threat for environment. To capture this impact EG and GS is taken into account, as evidenced by the literature, where EG and GS are assessed for the following possibilities:

1. $\beta_{3}$ = $\beta_{4}$=  0, represents an insignificant relationship between ED and EG.
2. $\beta_{3}$ > 0, $\beta_{4}$=  0, the economic growth has a positive impact on ED and insignificant GS means the economic growth is monotonically increasing.
3. $\beta_{3}$ < 0, $\beta_{4}$=  0, EG has a negative impact on ED and GS is insignificant mean the economic growth is monotonically decreasing.
4. $\beta_{3}$ > 0, $\beta_{4}$<  0, EG has adopting positive influence while the GS has implementing negative influence mean the inverted U-shape exists between ED and economic growth.
5.  $\beta_{3}$< 0, $\beta_{4}$ >  0, the EG has a negative impact while the GS has a positive impact means the U-shape relationship exists between ED and economic growth.

### 3.1 Data description

Ecological evaluation is important for sustainability and livings on the earth [5]. The rising environmental degradation is urging the researchers to identify the factors causing the pollution. The process of industrialization and extraction of natural resources take the significant importance [9] in both developed and developing countries [8]. SDGs of UN are significant for decisions and policy making globally and the SDGs are unachievable without improving the environmental concerns [5,9,12]. The process of industrialization and needs of the growing population is depleting the natural resources (as factors of production) and damaging the environment [17]. Environmental degradation is measured with two proxies CO2 and ecological footprints. CO2 is considered as weak indicator [18] and the ecological footprints envisage the pressure on natural environment [21] and briefly describes the environment degradation [22]. The economic activities urge to produce more and the industrialization is causing the environmental degradation thus the impact of economic activities is captured by taking the GDP growth. In this regard, EKC hypothesis is an important environment related economic theory which is proposed to be tested so the square of GDP growth is incorporated [35]. Financial development is considered an engine of growth [7,37] by reducing the cost of business. Machinery prices, private investment and many other aspects take the lead from financial development and damaging the environment thus the financial development is measured with domestic credit to private sector [2,7,50].

The study analyzed twenty years (2002–2021) data of 146 countries. The data of NR, FD, EG, GS are collected from the latest available World Development Indicators (WDI, https://databank.worldbank.org) and ED is collected from the global footprint network (GFN, www.footprintnetwork.org) [62].

Table 1 elaborates the abbreviation, measuring units and sources of the variables.

**Table 1:** Description of variables.

Variable Name	Abbreviation	Measuring unit	Source
Environmental Degradation	ED	Ecological footprint per person	GFN
Natural Resources	NR	Natural resource depletion (% of GNI)	WDI
Financial Development	FD	Domestic Credit to Private Sector by banks (% of GDP)	WDI
Economic Growth	EG	GDP growth (annual %)	WDI
Economic Growth Square	GS	Square of GDP growth (annual %)	WDI

Table 2 explains the cross frequency distribution of 2920 observations of 146 countries distributed into income groups and geographical regional groups. The 146 countries (S1 Table in S1 File) which are bifurcated region wise (S2 Table in S1 File) and with respect to income groups (S3 Table in S1 File) adopted from the World Bank.

**Table 2:** Frequency distribution of regions and income groups.

Regions	Income groups					
	High income	Low income	Lower middle income	Upper middle income	World	Total
East Asia & Pacific	120	0	160	120	0	400
Europe & Central Asia	480	0	60	200	0	740
Latin America & Caribbean	140	0	80	300	0	520
Middle East & North Africa	120	0	140	20	0	280
North America	20	0	0	0	0	20
South Asia	0	0	120	0	0	120
Sub-Saharan Africa	0	340	380	100	0	820
World	0	0	0	0	20	20
Total	880	340	940	740	20	2920

Table 3 presents the descriptive statistics of the variables incorporated in this study. The high values of standard deviation relative to the means showing the large variations of the balanced dataset.

**Table 3:** Descriptive statistics.

Variable	Obs	Mean	Std. Dev.	Min	Max
ED	2920	3.083	2.648	.35	16.08
NR	2920	4.424	6.807	0	49.46
FD	2920	48.287	40.226	0	254.55
EG	2920	3.484	4.718	-36.66	53.38
GS	2920	34.385	94.65	0	2849.62

Table 4 depicts the correlation matrix where none of the variables is problematically correlated with others.

**Table 4:** Matrix of correlations.

Variables	ED	NR	FD	EG	GS
ED	1.000				
NR	−0.053	1.000			
FD	0.416	−0.350	1.000		
EG	−0.062	0.120	−0.205	1.000	
GS	−0.040	0.141	−0.116	0.318	1.000

## 4. Results interpretation and discussion

This section explains some test for suitability of estimation methodology and afterward described the estimated results.

**Fig 2:**
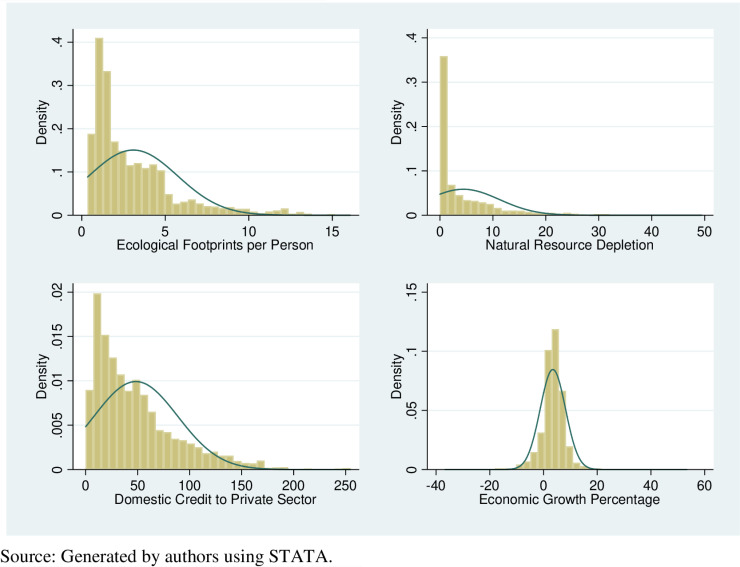
Normality of variables.

Table 5 presents the Shapiro–Francia W test and Fig. 2 shows the graph of variables to check the normality of data. The less than 5% probability values show that the data is not normally distributed (S4 Table in S1 File) which violates the key assumptions of the classical linear regression model. Keeping in view the heterogeneous nature of the data quantile regression best suits here.

**Table 5:** Shapiro-Francia W test for normal data.

Variable	Obs	W’	V’	Z	Prob > z
ED	2,920	0.808	340.516	14.290	0.000
NR	2,920	0.6950	541.952	15.429	0.000
FD	2,920	0.878	216.912	13.185	0.000
EG	2,920	0.873	225.250	13.277	0.000

**Fig 3:**
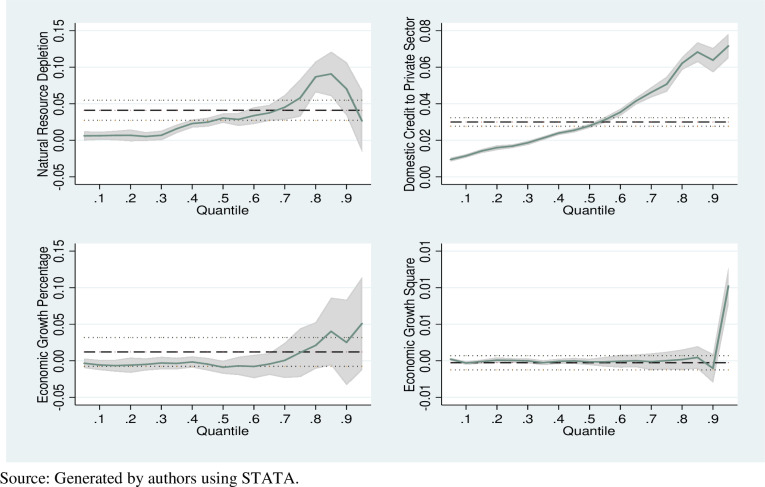
Comparative analysis of estimated parameter by quantile level.

Fig. 3 described that the technique incorporated in this study which briefs the estimated behavior of the skewed data (see Fig. 2 and Table 5) more accurate. The dotted line explains the confidence interval and estimated line using the OLS method while the highlighted area is the confidence interval and the line within the highlighted area shows the estimated line using quantile regression incorporated in this study. The OLS estimates the mean effect thus for NR it is overestimating till fifth quantile while underestimating with 7^th^ to 9^th^ quantile. Similarly, for FD the mean value is overestimating till 4^th^ quantile while underestimating from 6^th^ quantile to onward. Therefore, the incorporated methodology is performing better in explaining the behavior by briefly informing the various segments individually and overall behavior collectively.

Table 6 presents the estimated results of quantile regression by taking environment degradation as the dependent variable. The impact of natural resource depletion leads to environmental degradation whereas this impact varies in different quantiles. Initially, the impact of NR on ED is small which intensified with later quantiles till the 80^th^ quantile and declined afterward. The results show that the 10^th^, 40^th^ to 90^th^ quantiles are significant while the rest of the quantiles are positively associated but are insignificant. The estimated outcomes are according to the earlier the findings that overall rise in extraction of NR leads to ED [5,8,9,12,17]. The coefficients of FD in all the quantiles are significantly positive which state that the FD leads to unsustainability by promoting ED [63,64]. The impact is similar in direction and significant in all quantiles however the intensity varies among quantiles. The policy to regularize the FD for green growth seems suitable here. EG leads to reduce ED in initial quantiles while this impact becomes positive for 70^th^ to onward quantiles however the impact is only significant for the 99^th^ quantile with an ever larger coefficient. The estimated results suggest that the economies with low growth rates leads to reduce ED while the larger growth rates stimulating the ED. The impact of GS is negatively related to ED for the 10^th^, 30^th^ to 70^th^ quantiles with minute impact and positive for the rest of the quantiles. Whereas, it remains insignificant in all quantiles which does not validate the presence of EKC. Table 6 clarifies the roles of FD and NR leading toward unsustainability in all quantiles endorsing the negative impact of NR on ED for Brick countries [65], for G-11 countries [66] and for Pakistan [67]. This study endorsed the results [68] where FD is found to deteriorate ED. Furthermore, the geographical region wise estimation may clarify the directions for effective region wise policy insinuation.

**Table 6:** Global quantile regression taking environment degradation as dependent variable.

ED	10	20	30	40	50	60	70	80	90	99
NR	0.0063 *	0.0068	0.0068	0.0229***	0.0304***	0.0338***	0.0452***	0.0868***	0.0704**	0.0315
	(0.0032)	(0.0035)	(0.0041)	(0.0039)	(0.0055)	(0.0081)	(0.0103)	(0.0156)	(0.0235)	(0.0731)
FD	0.0115***	0.0160***	0.0186***	0.0239***	0.0279***	0.0354***	0.0463***	0.0621***	0.0638***	0.0516***
	(0.0005)	(0.0006)	(0.0007)	(0.0007)	(0.0009)	(0.0014)	(0.0017)	(0.0027)	(0.0040)	(0.0125)
EG	−0.0057	−0.0059	−0.0030	−0.0016	−0.0088	−0.0078	0.0006	0.0210	0.0252	0.245 *
	(0.0046)	(0.0050)	(0.0059)	(0.0057)	(0.0079)	(0.0116)	(0.0148)	(0.0224)	(0.0339)	(0.1050)
GS	−0.0003	0.0001	−0.0000	−0.0001	−0.0002	−0.0001	−0.0001	0.0002	−0.0010	0.0058
	(0.0002)	(0.0003)	(0.0003)	(0.0003)	(0.0004)	(0.0006)	(0.0007)	(0.0011)	(0.0017)	(0.0052)
Constant	0.520***	0.627***	0.767***	0.750***	0.847***	0.926***	0.919***	1.061***	2.907***	7.345***
	(0.0432)	(0.0473)	(0.0556)	(0.0536)	(0.0750)	(0.110)	(0.141)	(0.213)	(0.322)	(1.000)
N	2920	2920	2920	2920	2920	2920	2920	2920	2920	2920
Pseudo R^2^	0.08	0.1	0.13	0.15	0.16	0.16	0.13	0.12	0.15	0.12

Standard errors in parentheses, * p < 0.05, **p < 0.01, ***p < 0.001.

Table 7 explains the region wise impact of independent variables on ED. The detail of countries fall in these regions is provided (S2 Table in S1 File). East Asia and Pacific regions consist of 20 countries (including China, Japan, Malaysia, Singapore, Australia, New Zealand) where NR is leading to enhance ED. A unit (% of GNI) depletion of natural resources leads to degradation of ecology per person by 0.123 units and the estimated results are significant at less than 1% level. The increase in FD raises ED means the financial liberty is deteriorating the environmental sustainability. The EG and GS are negatively related to ED meaning the increase in income will lead to improving the environmental concerns in the region but the EKC hypothesis does not hold. Endorsing Nathaniel [69] which found adverse effect of natural resources and income (country wise) on environmental quality in ASEAN and Rahman & Alam [70] which estimated negative impact of FD on environment in Asia Pacific countries and validate the EKC which conflict with our results because of taking the dependent variable different. In this region the extraction of NR and FD should be linked with policy regularization to address the environmental concerns. The region includes the larger growing economies where an increase in income promotes the better environment. Europe and Central Asia consists of 37 countries (mainly Azerbaijan, Belgium, Denmark, France, Germany, Hungary, Russian Federation, Spain, Sweden, Switzerland, U.K, Turkey) the impact of NR is greater than East Asia & Pacific region while the FD is damaging the environment as well however less than East Asia & Pacific region. EG is insignificant while the GS is significantly negative in this region. Endorsing the finding of Afzal et al. [71] which estimated the negative impact of FD on four different variables of environment quality. The policy intervention to regularize the ecological impact of NR and FD is recommended for this region. Latin America and the Caribbean is a region of 26 countries (mainly Argentina, Brazil, Chile, Colombia, Mexico, Uruguay), the impact of NR is smaller while the impact of FD is comparatively larger than Europe and Central Asia. NR and FD lead to significantly degrade the environment similar with the findings of Jahanger et al. [10], while EG and GS is insignificant mean the income impact on the environment is unclear. The policy intervention to regularize the extraction of NR and amendment in FD policy is required to address the environmental concerns. Middle East and North Africa is a region of 14 countries (mainly Iran, Israel, Kuwait, Oman, Qatar, Saudi Arabia, U.A.E) where the impact of NR is significant and is greater than East Asia and Pacific region, Europe and Central Asia region, Latin America and Caribbean region. One-unit increase in NR will lead to increase ED by 0.226 units. The increase in FD significantly stimulate the environmental quality endorsing the findings of Nathaniel et al. [72] for Middle East and North African countries. Whereas the impact of income is insignificant/unclear. The policy intervention to regularize the extraction of NR and amendment in FD policy is required to address the environmental concerns. In North America (U.S.A) the effect of NR is significantly highest in the world where one unit of NR is lead to increase 2.82 units of ED. The impact of FD and EG is insignificant while the GS is negatively significant. Huang et al. [73] estimated similar finding for USA according to it FD and natural resources rent putting pressure on environment. The region is required to take concrete steps to make policies for NR those address the environmental concerns. In South Asia (Bangladesh, Bhutan, India, Nepal, Pakistan, Sri Lanka) the one-unit increase in NR is rising ED by 0.25 units which is second highest impact after North America. while the influence of FD and EG is insignificant while the impact of GS is significantly positive. Tahir et al. [74] found the negative impact of financial development on environment quality which differ due to time span and the carbon emission taken as dependent variable. The policy intervention for NR is required to deal ED. Sub Saharan Africa (main countries are Botswana, Burundi, Cameroon, Congo, Kenya, Madagascar, Nigeria, South Africa, Zimbabwe) is the region of 41 countries which shows that the FD the significantly positive relation with ED and insignificant for other independent factors. Thus the policy is required to deal the FD to address the ecological issues. The world (all countries) explains the significant positive impact of NR on ED. One-unit increase in NR will lead to degrade environment by 0.12 units. The impact of rest of the independent variables is unclear thus the policy intervention regarding NR is required to address the ED. The region wise analysis explains that NR impacts the environment adversely with the exception of Sub Saharan Africa and the FD is not improving the environment with the exception of North America and South Asia where the estimated results are insignificant/unclear and the validity of EKC is not confirmed in any region of the world.

**Table 7:** Regional quantile regression taking environment degradation as dependent variable.

ED	East Asia and Pacific	Europe and Central Asia	Latin America and Caribbean	Middle East and North Africa	North America	South Asia	Sub-Saharan Africa	World
NR	0.123***	0.148***	0.131***	0.226***	2.818 *	0.245 *	−0.00381	0.116**
	(0.0174)	(0.0290)	(0.0133)	(0.0347)	(1.005)	(0.111)	(0.00206)	(0.0356)
FD	0.0266***	0.00693**	0.0122***	0.0624***	0.0566	0.00106	0.00726***	0.00242
	(0.00220)	(0.00231)	(0.00285)	(0.0104)	(0.0888)	(0.00758)	(0.00106)	(0.00350)
EG	−0.0540 *	0.00664	0.00110	0.0263	0.157	−0.0705	−0.00363	0.0293
	(0.0259)	(0.0293)	(0.0121)	(0.0405)	(0.133)	(0.0501)	(0.00367)	(0.0144)
GS	−0.00657**	−0.00953***	0.000155	0.00180	−0.0956 *	0.0113 *	0.0000419	−0.00789 *
	(0.00247)	(0.00188)	(0.000609)	(0.00125)	(0.0337)	(0.00455)	(0.000205)	(0.00360)
Constant	0.904***	3.565***	1.441***	−1.779 *	4.671	0.514	1.066***	2.320***
	(0.230)	(0.238)	(0.142)	(0.774)	(4.568)	(0.413)	(0.0388)	(0.305)
N	400	740	520	280	20	120	820	20
Pseudo R^2^	0.18	0.31	0.07	0.23	0.5	0.15	0.03	0.51

Standard errors in parentheses, *  p < 0.05, ** p < 0.01, *** p < 0.001.

Table 8 presents the results of quantile regression taking into account the income groups of the countries (S3 Table in S1 File). The group of high-income countries shows that the NR is significantly enhancing ED. The estimated results show that a unit increase in NR will increase ED by 0.127 units per person. Endorsing the findings of Ali et al. [75] which states the negative impact of NR on environment in developed countries and contradicting the findings [64] which considered the G-7 countries only. The dynamics of G-7 countries may differ as this study is investigating the 44 high income countries. The impact of EG is significantly positive with ED and significant negative relation with GS confirms the presence of the EKC hypothesis by endorsing the earlier findings [76] for developed countries whereas the effect of FD is insignificant/unclear. The ecological issue needs to be addressed while incorporating the NR, and the estimated impact of economic growth in long run is not degrading the environment in these economies. The Upper Middle Income (37 countries) and the Lower Middle Income (47 countries) both groups behave similarly. These groups show that NR and FD are significantly increasing ED while the Upper Middle Income group contributing the more environment degradation compare with Lower Middle Income group. The impact of EG and GS remains insignificant/unclear. The policy action for both NR and FD is required to be incorporated to deal the environmental degradation in these economies. The Lower income group consist of 17 countries and the impact of NR, FD, EG and GS is insignificant/unclear. The world (global countries) shows that NR is increasing ED and GS is significantly negative. The EKC hypothesis is invalidated here endorsed the findings [39] for non-wealthy economies [77].

**Table 8:** Income group quantile regression taking environment degradation as dependent variable.

ED	High Income	Upper Middle Income	Lower Middle Income	Low Income	World
NR	0.127***	0.0339**	0.0161***	0.00170	0.116**
	(0.0148)	(0.0111)	(0.00240)	(0.00471)	(0.0356)
FD	0.00118	0.0101***	0.00672***	−0.00473	0.00242
	(0.00200)	(0.00210)	(0.000773)	(0.00498)	(0.00350)
EG	0.0550**	−0.00178	−0.00295	−0.00377	0.0293
	(0.0213)	(0.0123)	(0.00404)	(0.00728)	(0.0144)
GS	−0.00231 *	−0.000574	−0.000685	0.0000775	−0.00789 *
	(0.00110)	(0.000483)	(0.000361)	(0.000326)	(0.00360)
Constant	4.094***	1.634***	1.039***	1.014***	2.320***
	(0.215)	(0.128)	(0.0380)	(0.0983)	(0.305)
N	880	740	940	340	20
Pseudo R^2^	0.07	0.04	0.03	0.015	0.51

Standard errors in parentheses, *  p < 0.05, ** p < 0.01, *** p < 0.001.

S5 Table in S1 File explains the regional comparative outcome using regional dummies with bootstrap standard errors. North America is the reference region and all the estimation is considered relative to the base/reference region. All the regional coefficients are negative means that all the regions have less environmental degradation when compared with the base region that is North America. On average, a one-unit increase in NR will lead to a rise in ED by 0.0112 units. The intercept of base category is 7.284 while there exist significant differences in regional intercept. Comparing with base category South Asia keeps 7.269 units small intercept. Similarly, the estimated regional environmental degradation ranking (least to most) enlisted as South Asia, Sub Saharan Africa, Middle East and North Africa, East Asia and Pacific, Latin America and Caribbean, Europe and Central Asia, and North America. The ranking and slope vary with changing quantiles. S6 Table in S1 File explains the outcome of income group dummies and ranked the high income group as most the environmental degraded followed by Upper middle income, lower middle income and low income group with varying slopes as suggested [78].

## 5. Conclusions

The prevalent ecological deficit is devastating the ecosystem and is leading toward the environmental catastrophe by endangering the livings on earth. The natural resources depletion, financial development and the economic growth are tested to determine the driving factors of this unsustainability, the EKC hypothesis is evaluated to test the growth led environment pattern, across the global regions which are determined by the World Bank. This study incorporated twenty years (2002–2021) data of 146 countries. First, the countries are bifurcated into geographical regions and then into income wise groups to conduct this investigation. For normality of the data the Shapiro-Francia W test and graphical analysis were performed which proposed the heterogeneous nature of the data. The panel quantile regression is insensitive to heterogeneous data which is applied for this investigation. Firstly, the impact of dependent variables is tested on environment degradation across 146 countries using the 10^th^, 20^th^, 30^th^,40^th^, 50^th^, 60^th^, 70^th^, 80^th^, 90^th^ and 99^th^ quantiles. The estimated results suggest that the natural resources depletion is deteriorating the ecological footprints, which varies from smaller in initial quantiles to large in later quantiles and reverting again after 80^th^ quantile. However, the direction of the impact remained same. The effect of financial development is worsening the ecological footprints which remained smaller in initial quantiles and intensified in later quantiles with the exception after 90^th^ quantile. The deteriorating impact of FD on ED remained significant in all estimated quantiles. The insignificant impact of economic growth endorsed that the EKC hypothesis is not validated for the estimated quantiles, globally. This suggest policy intervention to restrict the natural resources depletion and binding the credit facilities to invest in ecosystem friendly projects by curtailing the process of ecological deficit for global sustainability which may be initiated from the most environment degraded quantiles. However, the pattern of economic growth led environmental concerns (EKC) remained undetermined. In all global geographical regions, natural resources and financial development are degrading the environment albeit with varying intensity. NR in North America and FD in the Middle East & North Africa are most severely deteriorating the ecology. The EKC is not validated for any region whereas the economic growth is improving the ecology in East Asia and the Pacific. By examining the income wise groups of countries, it is established that the negative impact of natural resources on ecology varies with income level. The financial development in upper middle income and lower middle income groups is significantly deteriorating the environment quality. However, the FD is improving the ecology in low income group but it is insignificant. The EKC hypothesis is established only in high income group.

### 5.1 Policy recommendations

i. Globally, the policy intervention is required to impose the restrictions on the extraction of naturally deteriorating resources to the extent of ensuring the presence of the regenerating capacity of the ecosystem.
ii. North American region and groups of High Income Countries suggested to be prioritized to take concrete steps to restrict the environmental degradation followed by the rest of the world.
iii. Suitable policies to restrict the environmental damages from the natural resources in North America and from financial development in Middle East & North Africa are required to be prioritized for sustainability.
iv. The governments should monitor and direct the financial institutions to disbursement of loans for research and development, green financing, efficient production which consume less resources and improves the environment quality. Financial development must be stapled with measures to improve the ecosystem without endangering the sustainability.
v. The role of economic growth is unclear with the exception of High Income group so it must be analyzed case to a case basis for further policy recommendation.

### 5.2. Future research and limitations

The study highlighted the behavior of the important variables to investigate the concerns of sustainability and environment quality by using the global heterogeneous data in various quantiles, bifurcating the global countries into income and region wise groups. Whereas the country level investigation may be useful for policy insinuation of a specific country. Furthermore, the incorporation of institutional quality and technological advancement to implement environmental policies may enhance the quality of the study.

### Supporting information

S1 File S1 Table: List of countries. S2 Table: Region wise list of countries. S3 Table: Income group of countries. S4 Table: Skewness/Kurtosis tests for Normality. S5 Table: Bootstrap quantile regression of Geographical regions by taking environment degradation as dependent variable. S6 Table: Bootstrap quantile regression of income groups by taking environment degradation as dependent variable. S7 Table: Quantile regression for panel data taking environment degradation as dependent variable.
(DOCX)
