# Correction: Ecological footprints, global sustainability, and the roles of natural resources, financial development, and economic growth

**DOI:** 10.1371/journal.pone.0323701

**Published:** 2025-05-08

**Authors:** Ali Hussein AL-Marshadi, Muhammad Aslam, Azhar Ali Janjua

The first author’s name is spelled incorrectly. The correct name is: Ali Hussein AL-Marshadi. The correct citation is: AL-Marshadi AH, Aslam M, Janjua AA (2025) Ecological footprints, global sustainability, and the roles of natural resources, financial development, and economic growth. PLoS ONE 20(3): e0317664. https://doi.org/10.1371/journal.pone.0317664.
