# Supplementary material for: Ecological footprints, global sustainability, and the roles of natural resources, financial development, and economic growth
Source: PLoS One. 2025 Mar 13;20(3):e0317664. doi: 10.1371/journal.pone.0317664 (PMC11906046; doi:10.1371/journal.pone.0317664)
Supplement: S1 File — S2 Table: Region wise list of countries. S3 Table: Income group of countries. S4 Table: Skewness/Kurtosis tests for Normality. S5 Table: Bootstrap quantile regression of Geographical regions by taking environment degradation as dependent variable. S6 Table: Bootstrap quantile regression of income groups by taking environment degradation as dependent variable. S7 Table: Quantile regression for panel data taking environment degradation as dependent variable. (DOCX) [file pone.0317664.s001.docx]

**Appendix**

| S1 Table: List of countries | | | | | | | |
| --- | --- | --- | --- | --- | --- | --- | --- |
| Sr. No. | Country | Sr. No. | Country | Sr. No. | Country | Sr. No. | Country |
| 1 | Albania | 38 | Cyprus | 75 | Korea, R. | 112 | Russian F. |
| 2 | Algeria | 39 | CÃƒÂ´te d'Iv. | 76 | Kuwait | 113 | Rwanda |
| 3 | Angola | 40 | Denmark | 77 | Kyrgyzstan | 114 | Samoa |
| 4 | Argentina | 41 | Dominica | 78 | Lebanon | 115 | Sao Tome |
| 5 | Armenia | 42 | Dominican | 79 | Lesotho | 116 | Saudi Arabia |
| 6 | Australia | 43 | Ecuador | 80 | Liberia | 117 | Senegal |
| 7 | Austria | 44 | Egypt | 81 | Luxembourg | 118 | Sierra Le. |
| 8 | Azerbaijan | 45 | El Salvador | 82 | Madagascar | 119 | Singapore |
| 9 | Bahamas | 46 | Eswatini | 83 | Malawi | 120 | Slovakia |
| 10 | Bangladesh | 47 | Fiji | 84 | Malaysia | 121 | Slovenia |
| 11 | Barbados | 48 | Finland | 85 | Mali | 122 | Solomon I. |
| 12 | Belarus | 49 | France | 86 | Mauritania | 123 | South Afr. |
| 13 | Belgium | 50 | Gabon | 87 | Mauritius | 124 | Spain |
| 14 | Belize | 51 | Gambia | 88 | Mexico | 125 | Sri Lanka |
| 15 | Benin | 52 | Georgia | 89 | Mongolia | 126 | Suriname |
| 16 | Bhutan | 53 | Germany | 90 | Morocco | 127 | Sweden |
| 17 | Bolivia | 54 | Ghana | 91 | Mozambique | 128 | Switzerland |
| 18 | Bosnia H. | 55 | Greece | 92 | Myanmar | 129 | Tajikistan |
| 19 | Botswana | 56 | Guatemala | 93 | Namibia | 130 | Tanzania, |
| 20 | Brazil | 57 | Guinea | 94 | Nepal | 131 | Thailand |
| 21 | Brunei D. | 58 | Guinea-B. | 95 | Netherlands | 132 | Togo |
| 22 | Bulgaria | 59 | Guyana | 96 | New Zeala. | 133 | Tonga |
| 23 | Burkina Faso | 60 | Haiti | 97 | Nicaragua | 134 | Trinidad T. |
| 24 | Burundi | 61 | Honduras | 98 | Niger | 135 | Tunisia |
| 25 | Cabo Verde | 62 | Hungary | 99 | Nigeria | 136 | Turkiye |
| 26 | Cambodia | 63 | India | 100 | Norway | 137 | Uganda |
| 27 | Cameroon | 64 | Indonesia | 101 | Oman | 138 | Ukraine |
| 28 | Central Afric | 65 | Iran, I | 102 | Pakistan | 139 | U.A.E |
| 29 | Chad | 66 | Iraq | 103 | Panama | 140 | U.K |
| 30 | Chile | 67 | Ireland | 104 | Papua N. | 141 | U.S.A |
| 31 | China | 68 | Israel | 105 | Paraguay | 142 | Uruguay |
| 32 | Colombia | 69 | Italy | 106 | Peru | 143 | Vanuatu |
| 33 | Comoros | 70 | Jamaica | 107 | Philippines | 144 | World |
| 34 | Congo | 71 | Japan | 108 | Poland | 145 | Zambia |
| 35 | Congo, D. | 72 | Jordan | 109 | Portugal | 146 | Zimbabwe |
| 36 | Costa Rica | 73 | Kazakhstan | 110 | Qatar |  |  |
| 37 | Croatia | 74 | Kenya | 111 | Romania |  |  |

S2 Table: Region wise list of countries

| **East Asia & Pacific** | **Europe & Central Asia** | **Sub-Saharan Africa** | **Latin America & Caribbean** |
| --- | --- | --- | --- |
| Australia | Albania | Angola | Argentina |
| Brunei D. | Armenia | Benin | Bahamas |
| Cambodia | Austria | Botswana | Barbados |
| China | Azerbaijan | Burkina Faso | Belize |
| Fiji | Belarus | Burundi | Bolivia |
| Indonesia | Belgium | Cabo Verde | Brazil |
| Japan | Bosnia | Cameroon | Chile |
| Korea, Republic | Bulgaria | Central African R. | Colombia |
| Malaysia | Croatia | Chad | Costa Rica |
| Mongolia | Cyprus | Comoros | Dominica |
| Myanmar | Denmark | Congo | Dominican Republic |
| New Zealand | Finland | Congo, Democratic | Ecuador |
| Papua New G. | France | CÃ´te d'Ivoire | El Salvador |
| Philippines | Georgia | Eswatini | Guatemala |
| Samoa | Germany | Gabon | Guyana |
| Singapore | Greece | Gambia | Haiti |
| Solomon Islands | Hungary | Ghana | Honduras |
| Thailand | Ireland | Guinea | Jamaica |
| Tonga | Italy | Guinea-Bissau | Mexico |
| Vanuatu | Kazakhstan | Kenya | Nicaragua |
| **Middle East & North Africa** | Kyrgyzstan | Lesotho | Panama |
| Algeria | Luxembourg | Liberia | Paraguay |
| Egypt | Netherlands | Madagascar | Peru |
| Iran | Norway | Malawi | Suriname |
| Iraq | Poland | Mali | Trinidad and Tobago |
| Israel | Portugal | Mauritania | Uruguay |
| Jordan | Romania | Mauritius | **South Asia** |
| Kuwait | Russian Federation | Mozambique | Bangladesh |
| Lebanon | Slovakia | Namibia | Bhutan |
| Morocco | Slovenia | Niger | India |
| Oman | Spain | Nigeria | Nepal |
| Qatar | Sweden | Rwanda | Pakistan |
| Saudi Arabia | Switzerland | Sao Tome | Sri Lanka |
| Tunisia | Tajikistan | Senegal |  |
| U.A.E | Turkiye | Sierra Leone |  |
| **North America** | Ukraine | South Africa |  |
| U.S.A | United Kingdom | Tanzania,Republic | |
|  |  | Togo |  |
|  |  | Uganda |  |
|  |  | Zambia |  |
|  |  | Zimbabwe |  |

S3 Table: Income group of countries

| **High income** | United Arab Emirates | **Lower middle income** | Tajikistan |
| --- | --- | --- | --- |
| Australia | United Kingdom | Algeria | Tanzania, United Republic |
| Austria | U.S.A | Angola | Tunisia |
| Bahamas | Uruguay | Bangladesh | Ukraine |
| Barbados | **Upper middle income** | Benin | Vanuatu |
| Belgium | Albania | Bhutan | Zambia |
| Brunei Darussalam | Argentina | Bolivia | Zimbabwe |
| Chile | Armenia | Cabo Verde | **Low income** |
| Croatia | Azerbaijan | Cambodia | Burkina Faso |
| Cyprus | Belarus | Cameroon | Burundi |
| Denmark | Belize | Comoros | Central African Republic |
| Finland | Bosnia and H. | Congo | Chad |
| France | Botswana | CÃ´te d'Ivoire | Congo, Democratic |
| Germany | Brazil | Egypt | Gambia |
| Greece | Bulgaria | Eswatini | Guinea-Bissau |
| Guyana | China | Ghana | Liberia |
| Hungary | Colombia | Guinea | Madagascar |
| Ireland | Costa Rica | Haiti | Malawi |
| Israel | Dominica | Honduras | Mali |
| Italy | Dominican Republic | India | Mozambique |
| Japan | Ecuador | Iran, Islamic R. | Niger |
| Korea, Republic of | El Salvador | Jordan | Rwanda |
| Kuwait | Fiji | Kenya | Sierra Leone |
| Luxembourg | Gabon | Kyrgyzstan | Togo |
| Netherlands | Georgia | Lebanon | Uganda |
| New Zealand | Guatemala | Lesotho |  |
| Norway | Indonesia | Mauritania |  |
| Oman | Iraq | Mongolia |  |
| Panama | Jamaica | Morocco |  |
| Poland | Kazakhstan | Myanmar |  |
| Portugal | Malaysia | Nepal |  |
| Qatar | Mauritius | Nicaragua |  |
| Romania | Mexico | Nigeria |  |
| Saudi Arabia | Namibia | Pakistan |  |
| Singapore | Paraguay | Papua New Guinea |  |
| Slovakia | Peru | Philippines |  |
| Slovenia | Russian Federation | Samoa |  |
| Spain | South Africa | Sao Tome and P. |  |
| Sweden | Suriname | Senegal |  |
| Switzerland | Thailand | Solomon Islands |  |
| Trinidad and Tobago | Tonga | Sri Lanka |  |
|  | Turkiye |  |  |

S4 Table: Skewness/Kurtosis tests for Normality

| Variable | Obs | Pr(Skewness) | | Pr(Kurtosis) | Prob>chi2 | |  |
| --- | --- | --- | --- | --- | --- | --- | --- |
| ED | 2,920 | 0.000 | 0.000 | | | 0.000 | |
| NR | 2,920 | 0.000 | 0.000 | | | 0.000 | |
| FD | 2,920 | 0.000 | 0.000 | | | 0.000 | |
| EG | 2,920 | 0.006 | 0.000 | | | 0.000 | |

S5 Table: Bootstrap quantile regression of Geographical regions by taking environment degradation as dependent variable

| ED | 20 | 40 | 60 | 80 | 99 |
| --- | --- | --- | --- | --- | --- |
| NR | 0.0112*** | 0.0175* | 0.0397*** | 0.0445*** | -0.0144 |
|  | (0.00211) | (0.00786) | (0.00423) | (0.00900) | (0.0139) |
| FD | 0.0126*** | 0.0115*** | 0.0142*** | 0.0304*** | 0.0223* |
|  | (0.00129) | (0.00140) | (0.00171) | (0.00458) | (0.0101) |
| EG | -0.00196 | -0.00271 | 0.00822 | 0.00699 | 0.0427* |
|  | (0.00358) | (0.00454) | (0.00617) | (0.0163) | (0.0186) |
| GS | -0.000652* | -0.000444 | -0.000730 | -0.000225 | -0.000167 |
|  | (0.000313) | (0.000546) | (0.000402) | (0.000883) | (0.00121) |
| East Asia and Pacific | -6.324*** | -6.190*** | -5.700*** | -3.718*** | 2.341*** |
|  | (0.199) | (0.233) | (0.546) | (0.527) | (0.700) |
| Europe and Central Asia | -6.025*** | -5.023*** | -4.583*** | -4.746*** | 1.050 |
|  | (0.188) | (0.212) | (0.501) | (0.290) | (0.692) |
| Latin America and Caribbean | -6.194*** | -6.067*** | -6.052*** | -5.688*** | 0.103 |
|  | (0.186) | (0.204) | (0.487) | (0.717) | (0.275) |
| Middle East and North Africa | -6.613*** | -6.613*** | -5.556*** | -3.733*** | 5.610*** |
|  | (0.175) | (0.243) | (0.528) | (0.370) | (0.607) |
| South Asia | -7.269*** | -7.285*** | -7.609*** | -7.959*** | -5.413*** |
|  | (0.187) | (0.215) | (0.478) | (0.757) | (0.382) |
| Sub Saharan Africa | -6.772*** | -6.817*** | -7.093*** | -7.232*** | -5.447*** |
|  | (0.184) | (0.219) | (0.474) | (0.358) | (0.174) |
| World | -5.724*** | -5.926*** | -6.430*** | -7.925*** | -7.823*** |
|  | (0.176) | (0.213) | (0.480) | (0.298) | (0.245) |
| Constant | 7.284*** | 7.620*** | 7.910*** | 8.138*** | 8.709*** |
|  | (0.199) | (0.218) | (0.479) | (0.397) | (0.563) |
| N | 2920 | 2920 | 2920 | 2920 | 2920 |
| Pseudo R2 | 0.1827 | 0.2332 | 0.256 | 0.2396 | 0.367 |
| Standard errors in parentheses, * p<0.05, ** p<0.01, *** p<0.001 | | | | | |

S6 Table: Bootstrap quantile regression of income groups by taking environment degradation as dependent variable

| ED | 20 | 40 | 60 | 80 | 99 |
| --- | --- | --- | --- | --- | --- |
| NR | 0.00756*** | 0.0226*** | 0.0408*** | 0.00933 | 0.0225 |
|  | (0.00137) | (0.00278) | (0.00532) | (0.00548) | (0.0257) |
| FD | 0.00113 | 0.00432*** | 0.00626*** | -0.00177 | -0.0175 |
|  | (0.000841) | (0.00126) | (0.000725) | (0.00288) | (0.0100) |
| EG | 0.00392 | 0.00211 | 0.00191 | 0.00940 | 0.0298 |
|  | (0.00303) | (0.00380) | (0.00402) | (0.0124) | (0.0221) |
| GS | -0.000212 | -0.000399* | -0.000541* | 0.000242 | 0.00224* |
|  | (0.000432) | (0.000171) | (0.000261) | (0.000821) | (0.00112) |
| Low Income | -2.519*** | -3.102*** | -3.638*** | -6.904*** | -13.29*** |
|  | (0.0859) | (0.119) | (0.0770) | (0.314) | (0.938) |
| Lower Middle Income | -2.272*** | -2.727*** | -3.259*** | -6.056*** | -6.189*** |
|  | (0.0755) | (0.111) | (0.0708) | (0.296) | (0.727) |
| Upper middle Income | -1.622*** | -1.988*** | -2.145*** | -3.950*** | -6.876*** |
|  | (0.0765) | (0.103) | (0.129) | (0.281) | (0.593) |
| World | -0.588*** | -1.418*** | -2.163*** | -5.330*** | -10.68*** |
|  | (0.0603) | (0.0947) | (0.0807) | (0.259) | (0.278) |
| Constant | 3.095*** | 3.696*** | 4.295*** | 8.188*** | 14.91*** |
|  | (0.0924) | (0.124) | (0.0778) | (0.332) | (1.054) |
| N | 2920 | 2920 | 2920 | 2920 | 2920 |
| Pseudo R2 | 0.2632 | 0.3116 | 0.3194 | 0.29 | 0.3466 |
| Standard errors in parentheses, * p<0.05, ** p<0.01, *** p<0.001 | | | | | |

**S7 Table:** Quantile regression for panel data taking environment degradation as dependent variable

| ED | 10 | 20 | 30 | 40 | 50 | 60 | 70 | 80 | 90 | 99 | |
| --- | --- | --- | --- | --- | --- | --- | --- | --- | --- | --- | --- |
| NR | 0.00240* | -0.00205* | 0.00943*** | 0.0178*** | 0.0287*** | -0.00002 | 0.0130*** | -0.0227 | 0.061*** | 0.064*** | |
|  | (0.00105) | (0.000864) | (0.000414) | (0.00108) | (0.00116) | (0.00973) | (0.00193) | (0.0206) | (0.0027) | (0.0190) | |
| FD | 0.00865*** | 0.0136*** | 0.0168*** | 0.0234*** | 0.0272*** | 0.0331*** | 0.0342*** | 0.0289** | 0.055*** | 0.046*** | |
|  | (0.000687) | (0.000400) | (0.000081) | (0.000056) | (0.0000900) | (0.00101) | (0.001) | (0.0101) | (0.0009) | (0.0021) | |
| EG | -0.000952 | 0.0110*** | -0.000576 | 0.00370*** | 0.00331*** | 0.00116 | 0.00466 | 0.014*** | -0.00506 | 0.170*** | |
|  | (0.00111) | (0.00105) | (0.000420) | (0.000560) | (0.000610) | (0.00131) | (0.00476) | (0.00327) | (0.00652) | (0.0359) | |
| GS | -0.000019 | -0.00073*** | -0.00020*** | -0.00035*** | -0.00026*** | 0.000105 | 0.000344 | -0.00041 | -0.0006* | 0.011*** | |
|  | (0.000071) | (0.00012) | (0.000020) | (0.000048) | (0.000019) | (0.00013) | (0.00018) | (0.00039) | (0.0003) | (0.0026) | |
| N | 2920 | 2920 | 2920 | 2920 | 2920 | 2920 | 2920 | 2920 | 2920 | 2920 | |
| Standard errors in parentheses, * p<0.05, ** p<0.01, *** p<0.001 | | | | | | | | | | |  |

S7 Table presents the estimated results following Adaptive Markov Chain Monte Carlo (MCMC) optimization described [6]. The NR is significantly stimulating ED for the 10^th^, 30^th^ to 50^th^, 70^th^, 90^th^ and 99^th^ quantiles. While significantly negative relation for the 20^th^ quantile and insignificant in the rest of the quantiles. FD is a significantly positive relation in all quantiles which mean financial development is deteriorating environment [3] however the intensity varies in all quantiles. EKC is significantly validated in the 20^th^, 40^th^ and 50^th^ quantiles.
